# Impact of finerenone on chronic kidney disease progression in Chinese patients with type 2 diabetes: a FIGARO-DKD subgroup analysis

**DOI:** 10.3389/fendo.2025.1568438

**Published:** 2025-04-30

**Authors:** Ping Li, Hongguang Zheng, Jianhua Ma, Weiping Lu, Ling Li, Fang Liu, Qing Su, Yuxiu Li, Yi Fang, Zhaohui Mo, Fei Xiong, Aiping Yin, Ying Zhang, Li Wang, Meike Brinker, Luke Roberts, Dalong Zhu

**Affiliations:** ^1^ Nanjing Drum Tower Hospital, The Affiliated Hospital of Nanjing University Medical School, Nanjing, China; ^2^ Nanjing Drum Tower Hospital, Xuzhou Medical University, Xuzhou, China; ^3^ General Hospital of Northern Theater Command, Shenyang, China; ^4^ Nanjing First Hospital, Nanjing, China; ^5^ Huaian No.1 People’s Hospital, Jiangsu, China; ^6^ Shengjing Hospital of China Medical University, Shenyang, China; ^7^ West China Hospital, Sichuan University, Chengdu, China; ^8^ Xinhua Hospital, Shanghai Jiaotong University School of Medicine, Shanghai, China; ^9^ Peking Union Medical College Hospital, Beijing, China; ^10^ 5th Medical Center of Chinese People’s Liberation Army General Hospital, Beijing, China; ^11^ The Third Xiangya Hospital of Central South University, Changsha, China; ^12^ Wuhan Hospital of Traditional Chinese and Western Medicine, Wuhan, China; ^13^ The First Affiliated Hospital of Xi’an Jiaotong University, Xi An, China; ^14^ The Third Affiliated Hospital of Guangzhou Medical University, Guangzhou, China; ^15^ Research and Development Beijing, Bayer Healthcare Company Limited, Beijing, China; ^16^ Cardiology and Nephrology Clinical Development, Bayer AG, Berlin, Germany; ^17^ Clinical Development, Bayer PLC, Reading, United Kingdom

**Keywords:** Chinese patients, chronic kidney disease, diabetic kidney disease, FIGARO-DKD, finerenone, type 2 diabetes

## Abstract

**Background:**

Type 2 diabetes (T2D) is a considerable and growing burden in the Chinese population, and affected adults are at high risk of developing chronic kidney disease (CKD). This subgroup analysis of the FIGARO-DKD trial explored the cardiovascular and kidney benefits of finerenone in Chinese patients with CKD and T2D on optimized renin–angiotensin system blockade.

**Methods:**

Patients with urine albumin-to-creatinine ratio (UACR) ≥30–<300 mg/g and estimated glomerular filtration rate (eGFR) ≥25–≤90 mL/min/1.73 m^2^, or UACR ≥300–≤5000 mg/g and eGFR ≥60 mL/min/1.73 m^2^, were randomized to finerenone or placebo. The primary cardiovascular composite outcome was time to cardiovascular death, non-fatal myocardial infarction, non-fatal stroke, or hospitalization for heart failure. The secondary kidney composite outcome was time to kidney failure, sustained eGFR decline ≥40% from baseline, or kidney-related death.

**Results:**

A total of 325 Chinese patients were included. Finerenone resulted in a numerical decrease in the risk of the cardiovascular composite outcome (hazard ratio 0.91; 95% confidence interval 0.50–1.67) and a significantly reduced risk of the key secondary kidney outcome (hazard ratio 0.48; 95% confidence interval 0.29–0.79; *p* = 0.0029). The incidence of investigator-reported hyperkalemia was high across both treatment arms. Nevertheless, the incidence of hyperkalemia leading to hospitalization and treatment discontinuation was low across treatment arms.

**Conclusions:**

Finerenone significantly reduced the composite kidney outcome, showed a trend to reduce cardiovascular outcomes, and demonstrated an acceptable safety profile in Chinese patients.

## 1 Introduction

Type 2 diabetes (T2D) is a global public health problem, affecting approximately 10.5% of the global population, or 536.6 million people in 2021 (1). Diabetes is increasingly prevalent, with approximately 141 million Chinese adults living with the condition, equating to approximately 11% of the population of China and >25% of the global T2D population (1–3). The increasing prevalence in China is attributable to an aging population and changing lifestyle factors, such as diet, alcohol consumption, smoking, and reduced physical activity (3, 4). Additionally, Asian patients with T2D are more than twice as likely to present with high albuminuria than White patients at diagnosis (5).

Data suggest that Asian patients with T2D have a higher prevalence of chronic kidney disease (CKD) than patients of other races and ethnicities (6, 7), and they tend to experience faster deterioration in their kidney function than White patients (6). In a recent study in China in patients with T2D, approximately 32% of patients had CKD (8). CKD is a common complication of T2D (9) that leads to kidney failure and a higher incidence of cardiovascular (CV) events in patients with any stage of CKD, with advanced CKD stages exhibiting the highest risk (10). CV disease (CVD) is a leading cause of death in patients with CKD (11). More than 25% of patients with CKD stages 1 or 2, and more than 50% of patients with end-stage kidney disease, die from CVD (12). Multimorbidity and advanced stages of CKD increase the risk of CVD, kidney complications, and mortality (10, 13–15).

These factors are significant when considering the Chinese population, as the incidence of CKD and progression to late-stage CKD is further exacerbated by an aging population, placing a heavy disease and economic burden on the Chinese healthcare system (16). This highlights an urgent need for new therapies, as well as early diagnosis and deployment of effective treatment interventions to reduce the burden of CKD in Chinese patients with T2D.

A bidirectional relationship between the heart and the kidneys has been described, whereby CKD can lead to progressively decreasing cardiac function and increased risk of CVD, and vice versa. The pathophysiology of both kidney disease and CVD is associated with mineralocorticoid receptor (MR) overactivation (17–19). Finerenone is a selective, nonsteroidal MR antagonist with demonstrated CV and kidney benefits in patients with CKD and T2D in two complementary phase 3 trials, where the combined patient populations covered the spectrum of CKD severity: FInerenone in reducing kiDnEy faiLure and dIsease prOgression in Diabetic Kidney Disease (FIDELIO-DKD, NCT02540993) and FInerenone in reducinG cArdiovascular moRtality and mOrbidity in Diabetic Kidney Disease (FIGARO-DKD, NCT02545049) (20).

The risk of CKD progression varies across different ethnicities (21). Therefore, it would be of interest to determine the impact of finerenone on slowing CKD progression and the reduction of CV risk in ethnically homogeneous populations. The objective of the present analysis is to take a closer look at the FIGARO-DKD (NCT02545049) trial data and evaluate the effect of finerenone on CV and kidney outcomes, as well as its safety, in Chinese patients with CKD and T2D.

## 2 Materials and methods

### 2.1 Study design and participants

The study design of the multicenter, randomized, double-blind, event-driven, phase 3 FIGARO-DKD trial has been described previously (20, 22). FIGARO-DKD was conducted in accordance with the principles of the Declaration of Helsinki, and the protocol was approved by relevant regulatory authorities and ethics committees for each trial site; written informed consent was obtained from all participants prior to their inclusion in the study (20). Eligible patients were aged ≥18 years with CKD and T2D and treated with optimized renin–angiotensin system blockade (20, 22). CKD was defined as urine albumin-to-creatinine ratio (UACR) ≥30–<300 mg/g and estimated glomerular filtration rate (eGFR) ≥25–≤90 mL/min/1.73 m^2^ (CKD stage 2–4), or UACR ≥300–≤5000 mg/g and eGFR ≥60 mL/min/1.73 m^2^ (CKD stage 1 or 2) (20, 22). Patients were required to have a serum potassium level ≤4.8 mmol/L at the time of screening (20, 22). Patients with symptomatic chronic heart failure with reduced ejection fraction were excluded (20, 22).

### 2.2 Subgroup of patients from China

In this analysis of the Chinese subgroup of the FIGARO-DKD trial, patients were exclusively enrolled from 67 sites across mainland China and were identified as being of Chinese race and ethnicity.

### 2.3 Trial procedures and outcomes

Following run-in, during which renin–angiotensin system therapy was optimized, patients were randomly assigned (1:1) to receive once-daily oral treatment with finerenone (10 mg or 20 mg) or matching placebo (20, 22). Patients with an eGFR at the screening visit of 25–<60 mL/min/1.73 m^2^ received an initial dose of 10 mg once daily, and those with eGFR ≥60 mL/min/1.73 m^2^ received an initial dose of 20 mg once daily. From month 1 onward, the target dose of finerenone or placebo was 20 mg once daily. After randomization, trial visits were conducted at month 1, month 4, and then every 4 months until trial completion.

The primary CV composite outcome was time to CV death, non-fatal myocardial infarction, non-fatal stroke, or hospitalization for heart failure (HHF). The first secondary kidney composite outcome was time to kidney failure, sustained eGFR decline ≥40% from baseline for ≥4 weeks, or kidney-related death. Further secondary outcomes included a kidney composite (kidney failure, sustained eGFR decline ≥57% from baseline for ≥4 weeks, or kidney-related death) and change in UACR to month 4 (20, 22). Safety analyses for FIGARO-DKD have been described previously (20). Briefly, they included assessment of adverse events (AEs) and central laboratory testing. AEs that occurred during the treatment period were designated as those that started or worsened during finerenone or placebo intake or up to 3 days after any temporary or permanent interruption.

### 2.4 Statistical analysis

This was a pre-specified analysis with a separate statistical analysis plan. Efficacy analyses were performed in all Chinese patients included in the full analysis set of the FIGARO-DKD trial, which included all randomized patients without critical Good Clinical Practice violations. Stratification factors were eGFR category at screening, albuminuria category at screening, and history of CVD.

In a time-to-event analyses, the superiority of finerenone over placebo was tested by means of stratified log-rank tests. Time-to-event treatment outcomes were expressed as hazard ratios (HRs) with corresponding confidence intervals (CIs). HRs (95% CI) are based on the stratified Cox proportional hazards model estimated within each level of the subgroup variable. Events were reported from randomization up to the end-of-trial visit. Patients without an event were censored at the date of their last contact, with complete information on all components of their respective outcomes. For the safety analysis, patients were included if they had undergone randomization, were without critical Good Clinical Practice violations, and had taken ≥1 dose of study drug or placebo (20, 22).

## 3 Results

### 3.1 Patients

Of the 740 patients from China in FIGARO-DKD at screening, 325 were randomized to receive finerenone (*n* = 162) or placebo (*n* = 163). All randomized patients completed the study and were included in the full analysis set. The demographics and baseline characteristics of patients in the finerenone and placebo arms were similar (**Table 1**). At baseline, patients had a mean age (± standard deviation) of 58.8 ± 11.0 years, a mean glycated hemoglobin level of 7.5% ± 1.4%, and a systolic blood pressure of 134.4 mmHg ± 14.6 mmHg. Mean eGFR at baseline was 76.46 mL/min/1.73 m^2^ and the median UACR was 742.12 mg/g, with approximately 80% of patients characterized as having UACR ≥300 mg/g and an eGFR ≥60 mL/min/1.73 m^2^. At baseline, 98.5% of patients received antidiabetic therapy; the most commonly used hypoglycemic agents were insulin and its analogs (73.8%) and biguanides (48.3%). The most commonly used non-antidiabetic agents at baseline were angiotensin receptor blockers (87.1%) and statins (49.8%). Diuretics were taken by 14.2% of patients. Only 3.1% and 0.3% of patients were treated with a glucagon-like peptide-1 receptor agonist and a sodium-glucose co-transporter-2 inhibitor, respectively. Approximately one-third of patients (32.6%) had a history of CVD, 51.7% had diabetic retinopathy, and the average duration of diabetes at baseline was 13.0 years. The median follow-up was 3.3 years, with patients receiving a mean daily dose of finerenone of 18.3 mg. A comparison of baseline characteristics between the Chinese subgroup and the overall FIGARO-DKD population is shown in **Supplementary Table S1**.

**Table 1:** Patient demographic and baseline characteristics.

	Chinese subgroup	
	Finerenone (*n* = 162)	Placebo (*n* = 163)
Age, years, mean ± SD	60.62 ± 10.20	56.98 ± 11.42
Sex, *n* (%)		
Female	42 (25.9)	34 (20.9)
Male	120 (74.1)	129 (79.1)
SBP, mmHg, mean ± SD	135.97 ± 13.16	132.84 ± 15.73
BMI, kg/m^2^, mean ± SD	26.51 ± 3.11	26.67 ± 3.35
Baseline waist-to-hip ratio, mean ± SD	0.96 ± 0.09	0.94 ± 0.06
Duration of diabetes, years, mean ± SD	13.34 ± 6.72	12.65 ± 7.06
HbA1c, %, mean ± SD	7.53 ± 1.41	7.53 ± 1.28
Serum potassium, mEq/L, arithmetic mean ± SD	4.19 ± 0.43	4.20 ± 0.37
eGFR, mL/min/1.73 m^2^, arithmetic mean ± SD	75.40 ± 18.32	77.50 ± 18.95
eGFR, mL/min/1.73 m^2^, *n* (%)		
<25	0	0
25–<45	10 (6.2)	6 (3.7)
45–<60	23 (14.2)	22 (13.5)
≥60	129 (79.6)	135 (82.8)
UACR, mg/g, median (IQR)	676 (320–1339)	779 (303–1614)
UACR, mg/g, *n* (%)		
<30	3 (1.9)	2 (1.2)
30–<300	35 (21.6)	38 (23.3)
≥300	124 (76.5)	123 (75.5)
Current smoker, *n* (%)	48 (29.6)	52 (31.9)
Medication use at baseline, *n* (%)		
ACE inhibitors	15 (9.3)	25 (15.3)
ARBs	145 (89.5)	138 (84.7)
Beta blockers	41 (25.3)	37 (22.7)
Diuretics	21 (13.0)	25 (15.3)
Statins	81 (50.0)	81 (49.7)
Potassium supplements	3 (1.9)	0
Potassium-lowering agents	0	0
Glucose-lowering therapies	161 (99.4)	159 (97.5)
Insulin and analogues	122 (75.3)	118 (72.4)
Sulfonylureas	25 (15.4)	27 (16.6)
DPP-4 inhibitors	16 (9.9)	19 (11.7)
GLP-1RAs	5 (3.1)	5 (3.1)
SGLT-2 inhibitors	1 (0.6)	0
Alpha glucosidase inhibitors	71 (43.8)	78 (47.9)
Medical history at baseline, *n* (%)		
History of CVD	56 (34.6)	50 (30.7)
Hypertension	151 (93.2)	147 (91.4)
Hyperlipidemia	89 (54.9)	101 (62.0)
Diabetic retinopathy	86 (53.1)	82 (50.3)
Coronary artery disease	39 (24.1)	51 (31.3)
Diabetic neuropathy	68 (42.0)	73 (44.8)
Myocardial infarction	7 (4.3)	8 (4.9)
Peripheral arterial occlusive disease	33 (20.4)	26 (16.0)
Ischemic stroke	36 (22.2)	26 (16.0)
Atrial fibrillation and atrial flutter	2 (1.2)	2 (1.2)
Heart failure	1 (0.6)	3 (1.8)
Percutaneous coronary intervention	8 (4.9)	14 (8.6)
Coronary artery bypass grafting	11 (6.8)	5 (3.1)
Periodontal disease	12 (7.4)	7 (4.3)
Internal carotid artery dissection	0	0

ACE, angiotensin-converting enzyme; ARB, angiotensin receptor blocker; BMI, body mass index; CVD, cardiovascular disease; DDP-4, dipeptidyl peptidase-4; eGFR, estimated glomerular filtration rate; GLP-1RA, glucagon-like peptide-1 receptor agonist; HbA1c, glycated hemoglobin; IQR, interquartile range; SBP, systolic blood pressure; SD, standard deviation; SGLT-2, sodium-glucose co-transporter-2; UACR, urine albumin-to-creatinine ratio.

### 3.2 Efficacy

#### 3.2.1 Cardiovascular outcomes

**Figure 1:**
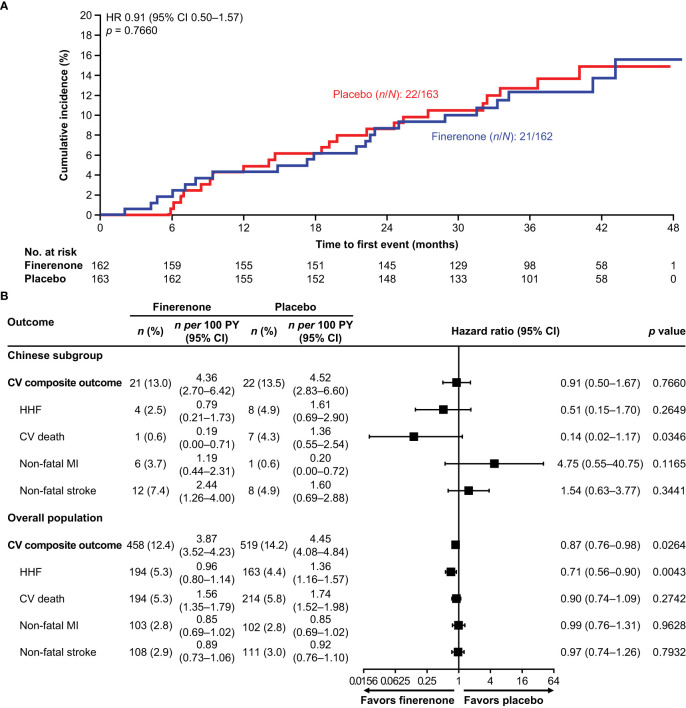
**(A)** Incidence of the primary CV composite outcome (time to first CV death, non-fatal stroke, non-fatal MI, and HHF) in the Chinese subgroup. **(B)** Incidence of the primary CV composite outcome (time to first CV death, non-fatal stroke, non-fatal MI, and HHF) and its components in the Chinese subgroup and the overall population. CI, confidence interval; CV, cardiovascular; HHF, hospitalization for heart failure; HR, hazard ratio; MI, myocardial infarction; PY, patient-years.

In the overall FIGARO-DKD population, the primary CV composite outcome occurred in 458/3686 (12.4%) patients in the finerenone arm and 519/3666 (14.2%) of patients in the placebo arm (HR 0.87; 95% CI 0.76–0.98; *p* = 0.03), with the benefit driven primarily by a lower incidence in HHF (HR 0.71; 95% CI 0.56–0.90) (20, 22). In the Chinese subgroup, the risk of the first occurrence of the primary CV composite outcome was numerically reduced in the finerenone arm versus the placebo arm (21 [13.0%] versus 22 [13.5%]; HR 0.91; 95% CI 0.50–1.67; *p* = 0.7660). The incidence of HHF was also numerically lower in the finerenone arm than in the placebo arm (4 [2.5%] versus 8 [4.9%]; HR 0.51; 95% CI 0.15–1.70; *p* = 0.2649), whereas the incidence of CV death was significantly lower in the finerenone arm than in the placebo arm (1 [0.6%] versus 7 [4.3%]; HR 0.14; 95% CI 0.02–1.17; *p* = 0.0346). Non-fatal myocardial infarction occurred in 6 (3.7%) patients treated with finerenone and 1 (0.6%) patient treated with placebo (HR 4.75; 95% CI 0.55–40.75; *p* = 0.1165). Non-fatal stroke occurred in 12 (7.4%) and 8 (4.9%) patients treated with finerenone and placebo, respectively (HR 1.54; 95% CI 0.63–3.77; *p* = 0.3441) (**Figures 1A, B**).

#### 3.2.2 Kidney outcomes

**Figure 2:**
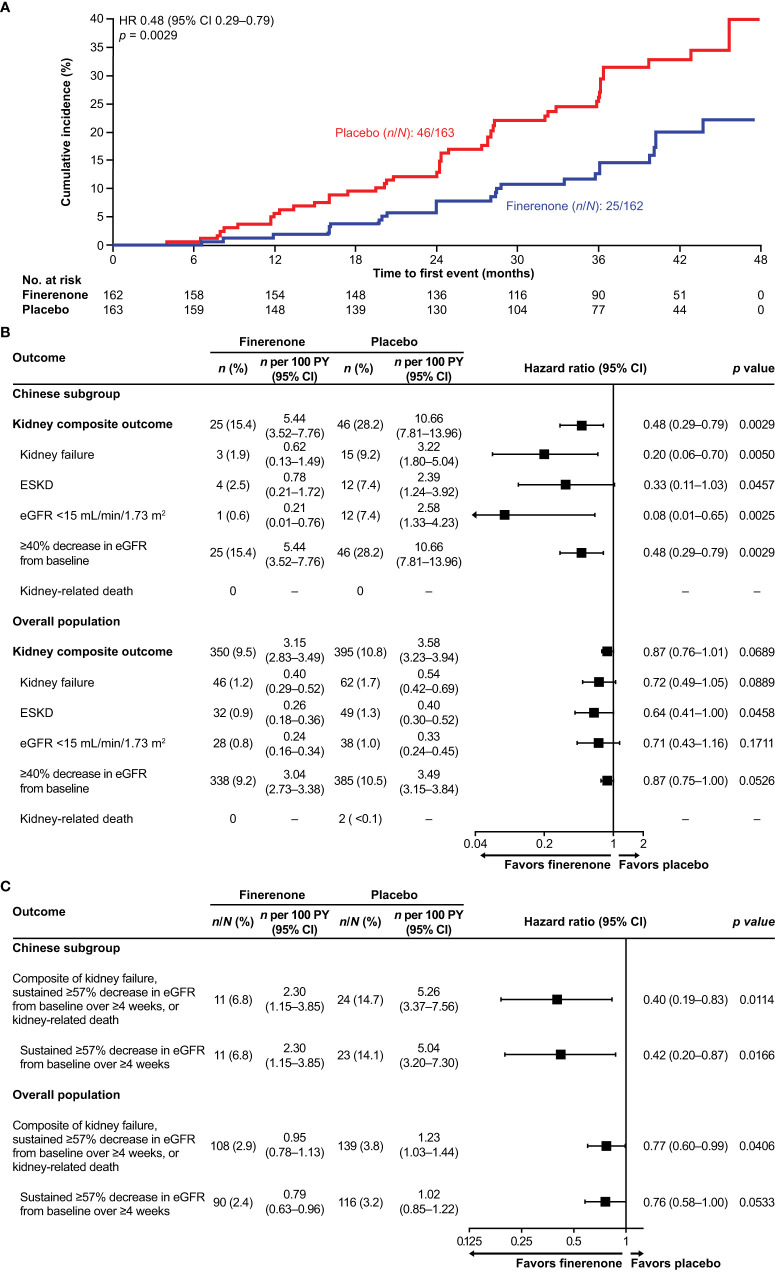
**(A)** Incidence of the key secondary kidney composite outcome (time to kidney failure, sustained eGFR decline ≥40% from baseline, or kidney-related death) in the Chinese subgroup. **(B)** Incidence of the key secondary kidney composite outcome (time to kidney failure, sustained ≥40% decrease in eGFR from baseline, or kidney-related death), and its components in the Chinese subgroup and the overall population. **(C)** Incidence of the additional secondary kidney composite outcome (time to kidney failure, sustained eGFR decline ≥57% from baseline over ≥4 weeks, or kidney-related death) and the sustained eGFR decline ≥57% component in the Chinese subgroup. CI, confidence interval; eGFR, estimated glomerular filtration rate; ESKD, end-stage kidney disease; HR, hazard ratio; PY, patient-years.

**Figure 3:**
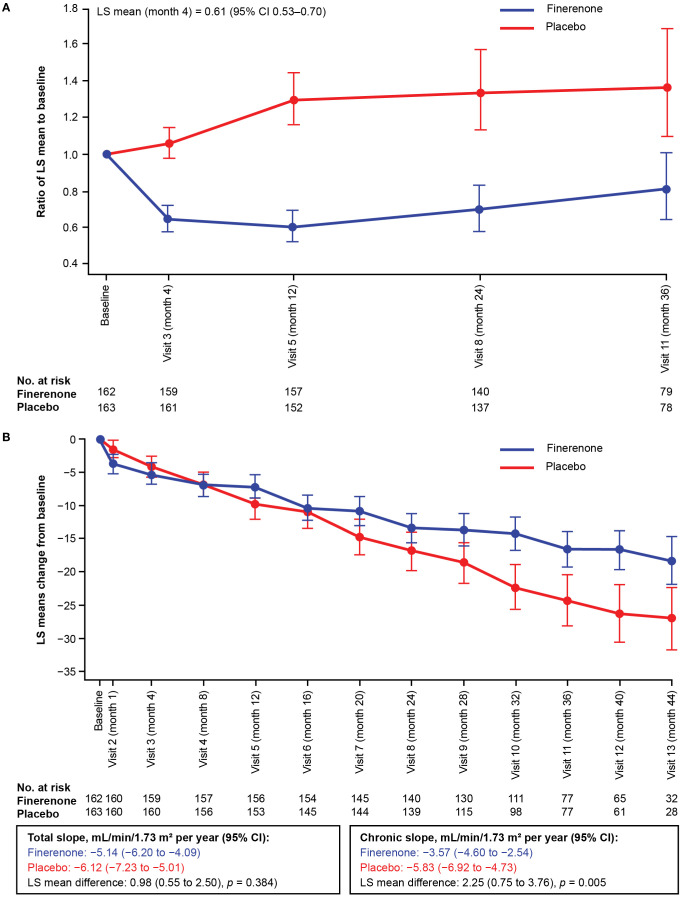
**(A)** Change in UACR from baseline in the Chinese subgroup over time. **(B)** Change in eGFR from baseline in the Chinese subgroup over time. The LS mean and 95% CI were derived from a mixed model with the following factors: treatment arm, region, screening eGFR category, screening albuminuria type, time, time to treatment and log-transition baseline value that matches the screen-phase albuminuria type; and with logarithmic-transformed baseline value × time as covariate. Independent unstructured covariance patterns were estimated for each treatment arm. This analysis excluded values after the date of end-stage kidney disease. CI, confidence interval; eGFR, estimated glomerular filtration rate; LS, least-squares; UACR, urine albumin-to-creatinine ratio.

In the overall FIGARO-DKD population, the secondary kidney composite outcome occurred in 350 (9.5%) patients in the finerenone arm and in 395 (10.8%) patients in the placebo arm (HR 0.87; 95% CI 0.76–1.01). In the Chinese subgroup, the key secondary kidney composite outcome was observed in 25 (15.4%) and 46 (28.2%) patients in the finerenone and placebo arms, respectively. Finerenone significantly reduced the risk of the key secondary kidney composite outcome (HR 0.48; 95% CI 0.29–0.79; *p* = 0.0029) and the kidney composite outcome of kidney failure, sustained eGFR decline ≥57% from baseline for ≥4 weeks or kidney-related death (HR 0.40; 95% CI 0.19–0.83; *p* = 0.0114; **Figures 2A–C**). At month 36, the number needed to treat to observe the benefits of finerenone on the kidney composite outcomes of ≥40% and ≥57% sustained eGFR decline was 7 (95% CI 4–22) and 12 (95% CI 6–60) patients, respectively. Reduction in UACR at month 4 was observed with finerenone versus placebo (ratio of least-squares [LS] means 0.61; 95% CI 0.53–0.70), and the geometric average UACR in the finerenone arm was consistently lower than that in the placebo arm at all study visits (**Figure 3A**).

Within the Chinese subgroup, treatment with finerenone significantly attenuated the annualized LS mean change in eGFR from month 4 to the end of treatment (chronic eGFR slope) compared with placebo. The chronic eGFR slope was −3.57 mL/min/1.73 m^2^ per year in the finerenone arm and −5.83 mL/min/1.73 m^2^ per year in the placebo arm (difference in LS means of 2.25 mL/min/1.73 m^2^; 95% CI 0.75–3.76). The annualized LS mean change in eGFR slope from baseline to end of treatment (total eGFR slope) was numerically lower in patients receiving finerenone (−5.14 mL/min/m^2^ per year) than those receiving placebo (−6.12 mL/min/m^2^ per year), resulting in a non-significant difference in LS means of 0.98 mL/min/1.73 m^2^ (95% CI −0.55 to 2.50; *p* = 0.3844). The total eGFR slope crossover occurred at month 8 (**Figure 3B**, **Supplementary Table S2**).

### 3.3 Safety profile

In the overall FIGARO-DKD population, the frequency of AEs did not differ substantially between treatment arms. In the Chinese subgroup, the incidence of treatment-emergent AEs was also similar between the finerenone (98.1%) and placebo (96.9%) arms (**Table 2**). The incidence of investigator-reported hyperkalemia in the Chinese subgroup was similar between the finerenone (19.1%) and placebo arms (18.5%). The proportion of patients in the Chinese subgroup among whom treatment-emergent hyperkalemia led to hospitalization or treatment discontinuation was low (finerenone: 0.6% versus placebo: 0% and finerenone: 0.6% versus placebo: 0.6%, respectively). Finerenone treatment was associated with a greater increase in serum potassium level from baseline than placebo. The mean change in serum potassium level from baseline at month 1 in the Chinese subgroup was 0.134 mmol/L and 0.011 mmol/L in the finerenone and placebo arms, respectively, with a difference between the two arms of ∼0.123 mmol/L. The proportion of patients in the Chinese subgroup with a serum potassium level >5.5 mmol/L at any time during treatment was similar between the finerenone (9.3%) and placebo (8.7%) arms. More patients with a serum potassium level >6.0 mmol/L were treated with finerenone (5.0%) than placebo (2.5%) (**Table 2**).

**Table 2:** TEAEs and hyperkalemia events.

*n* (%)	Chinese subgroup	
	Finerenone (*n* = 162)	Placebo (*n* = 162)
Any TEAE^†^	159 (98.1)	157 (96.9)
Any TEAE leading to study drug discontinuation	8 (4.9)	8 (4.9)
Any SAE	82 (50.6)	84 (51.9)
Any SAE leading to study drug discontinuation	3 (1.9)	4 (2.5)
AE with outcome death	2 (1.2)	1 (0.6)
Any hyperkalemia TEAE	31 (19.1)	30 (18.5)
Any hyperkalemia leading to discontinuation of study drug	1 (0.6)	1 (0.6)
Any hyperkalemia leading to hospitalization	1 (0.6)	0
Any serious hyperkalemia	1 (0.6)	1 (0.6)
Any serious hyperkalemia reported as life-threatening	0	1 (0.6)
Hyperkalemia with outcome death	0	0
Serum potassium^‡^ (mmol/L)		
>5.5	15/161 (9.3)	14/161 (8.7)
>6.0	8/161 (5.0)	4/161 (2.5)

^†^This category contains any AEs reported during the study, including AEs that may occur between random grouping and first administration of the study drug in patients who did not receive their first dose on the day of random grouping. ^‡^The number of patients at risk with ≥1 laboratory assessment meeting the criteria during the treatment. For evaluation during treatment, only evaluation from start of treatment to 3 days after temporary interruption or permanent discontinuation of study drug is considered. AE, adverse event; SAE, serious adverse event; TEAE, treatment-emergent adverse event.

## 4 Discussion

The benefits of finerenone on CV and kidney outcomes in patients with T2D and CKD (stage 2–4 CKD with moderately elevated albuminuria or stage 1 or 2 CKD with severely elevated albuminuria demonstrated numerically) have been previously reported in the FIGARO-DKD trial (20). To assess for the benefits of finerenone in Chinese patients, we conducted this prespecified subgroup analysis in Chinese participants enrolled in the FIGARO-DKD trial.

In Chinese patients with T2D and CKD, finerenone numerically reduced the risk of the primary CV composite outcome compared with placebo. However, the relative risk reduction for this outcome was not statistically significant, unlike in the overall FIGARO-DKD population (13% reduction [95% CI 0.76–0.98]; *p* = 0.03). Reductions in CV death (significant in the Chinese population) and HHF were the main drivers for both the trend towards CV benefit in the Chinese subgroup and the observed CV benefit in the overall FIGARO-DKD population. This may have been due to the low number of events across both treatment arms in the Chinese subgroup. Compared with the overall FIGARO-DKD population, fewer patients in the Chinese subgroup had a history of CVD or heart failure at baseline, which may help explain this finding (20).

With regard to kidney outcomes, finerenone demonstrated a clinically significant benefit versus placebo in reducing the risk of the first secondary kidney composite outcome (time to kidney failure, sustained eGFR decline ≥40% from baseline for ≥4 weeks, or kidney-related death) in the Chinese subgroup. This is broadly consistent with data from the overall FIGARO-DKD population, where a reduction in the risk of the secondary kidney outcome was also observed with finerenone versus placebo; however, statistical significance was not achieved in the overall FIGARO-DKD population (20). The results reported in this subgroup analysis suggest an early and sustained benefit with finerenone among Chinese patients. A clinically significant benefit was also observed for the eGFR ≥57% secondary kidney composite outcome in both the overall FIGARO-DKD population and the Chinese subgroup. As surrogate endpoints, a ≥40% decline in eGFR is associated with kidney failure and mortality, whereas a ≥57% decline is a clearer indicator of kidney failure because it is a later event in CKD progression (23, 24). These results provide further evidence of the beneficial effects of finerenone in slowing CKD progression.

The data from this analysis showed that patients in the Chinese subgroup had higher composite kidney outcome event rates than patients in the overall FIGARO-DKD population in each treatment arm (finerenone: 5.44 [95% CI 3.52–7.76] per 100 patient-years [PY] versus 3.15 [95% CI 2.83–3.49] per 100 PY; placebo: 10.66 [95% CI 7.81–13.96] per 100 PY versus 3.58 [95% CI 3.23–3.94] per 100 PY, respectively). Event rates were higher despite approximately 80% of Chinese patients having baseline eGFR ≥60 mL/min/1.73 m^2^. This may be attributable to the higher levels of albuminuria at baseline observed in the Chinese subgroup versus the overall FIGARO-DKD population (baseline median UACR 742.12 mg/g versus 308.18 mg/g, respectively) being the key driver for disease progression (20). This trend of increased kidney event rates was also observed in the overall FIGARO-DKD population for patients with baseline UACR ≥300 mg/g (22). Because albuminuria is predictive of CV and kidney events (25), this further highlights guideline recommendations for regular UACR screening for all people with T2D (26), supporting early intervention to improve outcomes in the under-recognized patient population with high CV risk and among those who are at risk despite having preserved kidney function. In addition to albuminuria, the percentage of patients with baseline diabetic retinopathy, a marker for microvascular complications shown to be associated with advanced risk for end-stage kidney disease, was also higher in the Chinese subgroup compared with the overall FIGARO-DKD population (52% versus 31%, respectively) (27).

Consistent with the data reported for the overall FIGARO-DKD population in patients with baseline UACR ≥300 mg/g, finerenone demonstrated a greater reduction in the risk of kidney outcomes in the Chinese subgroup (22). Furthermore, the observed reduction in UACR with finerenone in the Chinese subgroup (39% placebo-corrected reduction from baseline to month 4) is slightly higher than the 32% reported for the overall FIGARO-DKD population (20). This is noteworthy as a reduction in UACR by ≥30% is recommended by the American Diabetes Association to slow CKD progression in individuals with UACR ≥300 mg/g (24, 28). In addition, and similar to that reported for the overall FIGARO-DKD population, the total eGFR slope data showed improvement of kidney function with finerenone versus placebo in the Chinese subgroup. Moreover, there was an earlier crossover of the finerenone eGFR slope with placebo in the Chinese subgroup compared with the overall FIGARO-DKD population, suggesting an early effect in slowing down kidney disease progression in a subgroup in which the disease appeared to progress faster.

MR overactivation leads to inflammation and fibrosis, contributing to CKD progression and CV events; finerenone exerts its effects by targeting this overactivation (19). Additive benefits may be observed when finerenone is combined with renin–angiotensin system inhibitors, sodium-glucose co-transporter-2 inhibitors, and glucagon-like peptide-1 receptor agonists as recommended in international guidelines (26). These drug classes have distinct but complementary effects, which together may help address CKD via different pathways targeting inflammation, hemodynamic dysfunction, and metabolic dysregulation (29). However, to the best of our knowledge, there are currently no reported data on the effects of this combination in the Chinese population.

Safety findings with finerenone in the Chinese subgroup were generally consistent with those observed in the overall FIGARO-DKD population. Hyperkalemia is a common class AE associated with the mechanism of action of finerenone and can be managed by routine potassium monitoring (19, 30, 31). In the Chinese subgroup, the incidence of investigator-reported hyperkalemia was comparable between the finerenone and placebo arms, indicating that the relative risk of hyperkalemia was not substantially increased with finerenone treatment in this subgroup. However, compared with the overall FIGARO-DKD population, investigator−reported hyperkalemia incidence rates were higher in the Chinese subgroup for both treatment arms (19.1% vs 10.8% with finerenone and 18.5% vs 5.3% with placebo in the Chinese subgroup and overall FIGARO-DKD population, respectively) (20). In contrast, the proportion of patients with a central laboratory serum potassium level >5.5 mmol/L in the Chinese subgroup (finerenone, 9.3%; placebo, 8.7%) was similar to that reported in the overall FIGARO-DKD population (finerenone, 13.5%; placebo 6.4%) (20). These results suggest that the serum potassium level threshold for investigator-reported hyperkalemia may have been lower in China compared with other regions where the FIGARO-DKD trial was conducted based on local guidelines, leading to some over-reporting. These guidelines report higher frequencies of hyperkalemia in Asia than in Europe or North America due to variations in healthcare systems and local clinical practice (32). Nevertheless, the incidence of clinically meaningful events was low in the Chinese subgroup, with few hospitalizations or study drug discontinuations due to hyperkalemia and no hyperkalemia-related deaths; findings that are similar to those in the overall FIGARO-DKD population (20).

Results from the FIDELIO-DKD trial (a complementary trial to FIGARO-DKD mainly including patients with stage 3–4 CKD) and a pre-specified analysis in the Chinese subgroup of FIDELIO-DKD demonstrated that finerenone reduced the kidney and CV composite outcomes versus placebo, with a similar safety profile across subgroups (33, 34). The current analysis complements the previously published results on Chinese patients with mostly CKD stage 3–4 by demonstrating the CV and kidney benefits with finerenone in Chinese patients with CKD stage 2–4 and moderately elevated albuminuria, or CKD stage 1 or 2 and severely increased albuminuria – two patient populations at high risk of CVD that have been understudied in previous trials.

One limitation of this study was the small population size included in the subgroup analysis. This may have resulted in the broad CIs observed in some of the CV and kidney component data. In addition, there were a limited number of CV events in this population during the trial, which may have contributed to the non-significant reduction in CV risk seen with finerenone. Because of these limitations, the results reported here should be considered exploratory. A further limitation is that the FIGARO-DKD enrollment criteria may not have captured the variety of T2D phenotypes in China, which have been reported to be associated with distinct cardiorenal risk profiles (35). As such, the patients selected in this analysis may not be representative of all Chinese people with CKD and T2D.

In summary, finerenone demonstrated clinically significant kidney benefits and suggested CV benefits in Chinese patients, broadly similar to the overall FIGARO-DKD population, with a low incidence of clinically meaningful hyperkalemia events. Data also emphasize the need for albuminuria screening in patients with T2D to ensure early treatment intervention especially when eGFR is still preserved to reduce the risk of CV events and CKD progression in albuminuric patients.

## Data Availability

Availability of the data underlying this publication will be determined according to Bayer’s commitment to the EFPIA/PhRMA ‘Principles for responsible clinical trial data sharing’. This pertains to scope, timepoint, and process of data access. As such, Bayer commits to sharing upon request from qualified scientific and medical researchers patient-level clinical trial data, study-level clinical trial data, and protocols from clinical trials in patients for medicines and indications approved in the US and EU as necessary for conducting legitimate research. This applies to data on new medicines and indications that have been approved by the EU and US regulatory agencies on or after January 1, 2014. Interested researchers can use www.vivli.org to request access to anonymized patient-level data and supporting documents from clinical studies to conduct further research that can help advance medical science or improve patient care. Information on the Bayer criteria for listing studies and other relevant information is provided in the member section of the portal. Data access will be granted to anonymized patient-level data, protocols, and clinical study reports after approval by an independent scientific review panel. Bayer is not involved in the decisions made by the independent review panel. Bayer will take all necessary measures to ensure that patient privacy is safeguarded.
