# Supplementary material for: Impact of finerenone on chronic kidney disease progression in Chinese patients with type 2 diabetes: a FIGARO-DKD subgroup analysis
Source: Front Endocrinol (Lausanne). 2025 Apr 30;16:1568438. doi: 10.3389/fendo.2025.1568438 (PMC12074935; doi:10.3389/fendo.2025.1568438)
Supplement: Supplementary file 1 [file DataSheet1.docx]

Supplementary Material

# Graphical abstract


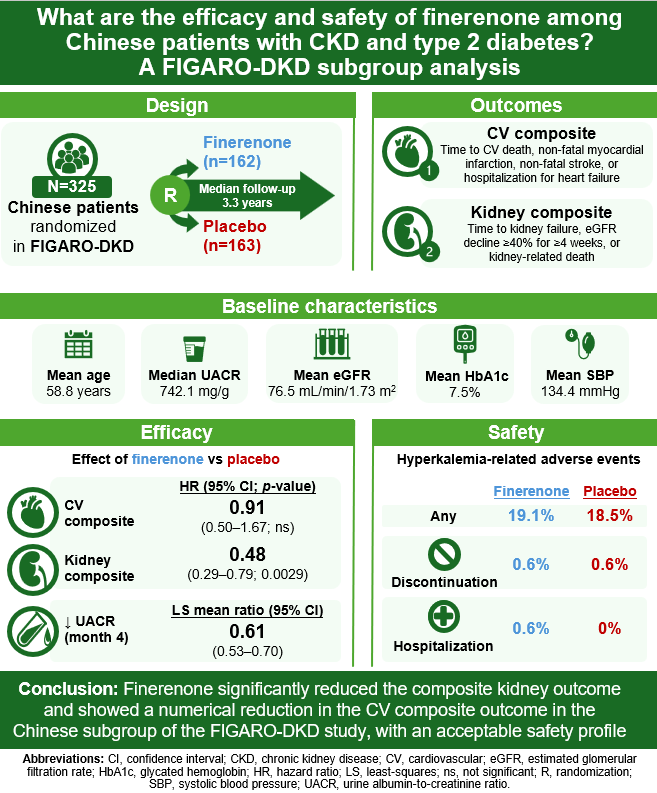


# Supplementary Tables

**Table S1.** Patient demographic and baseline characteristics in FIGARO-DKD: Chinese subgroup versus overall population

| **Characteristic** | **Chinese subgroup** | | **Overall population** | |
| --- | --- | --- | --- | --- |
|  | **Finerenone**  **(*n* = 162)** | **Placebo**  **(*n* = 163)** | **Finerenone**  **(*N* = 3686)** | **Placebo**  **(*N* = 3666)** |
| Age, year, mean ± SD | 60.62 ± 10.20 | 56.98 ± 11.42 | 64.13 ± 9.67 | 64.13 ± 10.00 |
| Sex, *n* (%) |  |  |  |  |
| Female | 42 (25.9) | 34 (20.9) | 1158 (31.4) | 1089 (29.7) |
| Male | 120 (74.1) | 129 (79.1) | 2528 (68.6) | 2577 (70.3) |
| SBP, mmHg, mean ± SD | 135.97 ± 13.16 | 132.84 ± 15.73 | 135.81 ± 13.96 | 135.70 ± 14.06 |
| BMI, kg/m^2^, mean ± SD | 26.51 ± 3.11 | 26.67 ± 3.35 | 31.46 ± 6.04 | 31.40 ± 5.93 |
| Baseline waist-to-hip ratio, mean ± SD | 0.96 ± 0.09 | 0.94 ± 0.06 | 1.00 ± 0.11 | 1.00 ± 0.11 |
| Duration of diabetes, years, mean ± SD | 13.34 ± 6.72 | 12.65 ± 7.06 | 14.53 ± 8.60 | 14.44 ± 8.44 |
| HbA1c, %, mean ± SD | 7.53 ± 1.41 | 7.53 ± 1.28 | 7.74 ± 1.39 | 7.69 ± 1.35 |
| Serum potassium, mEq/L, arithmetic mean ± SD | 4.19 ± 0.43 | 4.20 ± 0.37 | 4.33 ± 0.43 | 4.33 ± 0.43 |
| eGFR, mL/min/1.73 m^2^, arithmetic mean ± SD | 75.40 ± 18.32 | 77.50 ± 18.95 | 67.62 ± 21.65 | 67.99 ± 21.74 |
| eGFR, mL/min/1.73 m^2^, *n* (%) |  |  |  |  |
| <25 | 0 | 0 | 15 (0.4) | 12 (0.3) |
| 25–<45 | 10 (6.2) | 6 (3.7) | 641 (17.4) | 610 (16.6) |
| 45–<60 | 23 (14.2) | 22 (13.5) | 745 (20.2) | 789 (21.5) |
| ≥60 | 129 (79.6) | 135 (82.8) | 2285 (62.0) | 2254 (61.5) |
| UACR, mg/g, median (IQR) | 676 (320–1339) | 779 (303–1614) | 302 (105–749) | 315 (111–731) |
| UACR, mg/g, *n* (%) |  |  |  |  |
| <30 | 3 (1.9) | 2 (1.2) | 109 (3.0) | 98 (2.7) |
| 30–<300 | 35 (21.6) | 38 (23.3) | 1726 (46.8) | 1688 (46.0) |
| ≥300 | 124 (76.5) | 123 (75.5) | 1851 (50.2) | 1878 (51.2) |
| Current smoker, *n* (%) | 48 (29.6) | 52 (31.9) | 651 (17.7) | 636 (17.3) |
| Medication use at baseline, *n* (%) |  |  |  |  |
| ACE inhibitors | 15 (9.3) | 25 (15.3) | 2108 (57.2) | 2104 (57.4) |
| ARBs | 145 (89.5) | 138 (84.7) | 1576 (42.8) | 1561 (42.6) |
| Beta blockers | 41 (25.3) | 37 (22.7) | 1774 (48.1) | 1762 (48.1) |
| Diuretics | 21 (13.0) | 25 (15.3) | 1748 (47.4) | 1748 (47.7) |
| Statins | 81 (50.0) | 81 (49.7) | 2552 (69.2) | 2632 (71.8) |
| Potassium supplements | 3 (1.9) | 0 | 111 (3.0) | 104 (2.8) |
| Potassium-lowering agents | 0 | 0 | 24 (0.7) | 22 (0.6) |
| Glucose-lowering therapies | 161 (99.4) | 159 (97.5) | 3607 (97.9) | 3589 (97.9) |
| Insulin and analogues | 122 (75.3) | 118 (72.4) | 2023 (54.9) | 1970 (53.7) |
| Sulfonylureas | 25 (15.4) | 27 (16.6) | 1037 (28.1) | 1025 (28.0) |
| DPP-4 inhibitors | 16 (9.9) | 19 (11.7) | 896 (24.3) | 860 (23.5) |
| GLP-1RAs | 5 (3.1) | 5 (3.1) | 308 (8.4) | 242 (6.6) |
| SGLT-2 inhibitors | 1 (0.6) | 0 | 314 (8.5) | 304 (8.3) |
| Alpha glucosidase inhibitors | 71 (43.8) | 78 (47.9) | 160 (4.3) | 172 (4.7) |
| Medical history at baseline, *n* (%) |  |  |  |  |
| History of CVD | 56 (34.6) | 50 (30.7) | 1676 (45.5) | 1654 (45.1) |
| Hypertension | 151 (93.2) | 147 (91.4) | 3544 (96.1) | 3517(95.9) |
| Hyperlipidemia | 89 (54.9) | 101 (62.0) | 1515 (41.1) | 1550 (42.3) |
| Diabetic retinopathy | 86 (53.1) | 82 (50.3) | 1193 (32.4) | 1098 (30.0) |
| Coronary artery disease | 39 (24.1) | 51 (31.3) | 1148 (31.1) | 1147(31.3) |
| Diabetic neuropathy | 68 (42.0) | 73 (44.8) | 1046 (28.4) | 990 (27.0) |
| Myocardial infarction | 7 (4.3) | 8 (4.9) | 640 (17.4) | 616 (16.8) |
| Peripheral arterial occlusive disease | 33 (20.4) | 26 (16.0) | 587 (15.9) | 575 (15.7) |
| Ischemic stroke | 36 (22.2) | 26 (16.0) | 442 (12.0) | 425 (11.6) |
| Atrial fibrillation and atrial flutter | 2 (1.2) | 2 (1.2) | 328 (8.9) | 317(8.6) |
| Heart failure | 1 (0.6) | 3 (1.8) | 290 (7.9) | 281 (7.7) |
| Percutaneous coronary intervention | 8 (4.9) | 14 (8.6) | 217 (5.9) | 198 (5.4) |
| Coronary artery bypass grafting | 11 (6.8) | 5 (3.1) | 207 (5.6) | 199 (5.4) |
| Periodontal disease | 12 (7.4) | 7 (4.3) | 195 (5.3) | 174 (4.7) |
| Internal carotid artery dissection | 0 | 0 | 43 (1.2) | 50 (1.4) |

ACE, angiotensin-converting enzyme; ARB, angiotensin receptor blocker; BMI, body mass index; CVD, cardiovascular disease; DDP-4, dipeptidyl peptidase-4; eGFR, estimated glomerular filtration rate; GLP-1RA, glucagon-like peptide-1 receptor agonist; HbA1c, glycated hemoglobin; IQR, interquartile range; SBP, systolic blood pressure; SD, standard deviation; SGLT-2, sodium-glucose co-transporter-2; UACR, urine albumin-to-creatinine ratio.

**Table S2.** Total and chronic eGFR slopes for the Chinese subgroup

|  | **Treatment group (*n*)** | **LS means** | **95% CI** | ***p* value** | **Between-group difference** | **95% CI** |
| --- | --- | --- | --- | --- | --- | --- |
| **Total slope** | **Finerenone (*n* = 139)** | −5.14 | (−6.20 to −4.09) | 0.3844 | 0.98 | (−0.55 to 2.50) |
|  | **Placebo (*n* = 125)** | −6.12 | (−7.23 to −5.01) |  |  |  |
| **Chronic slope** | **Finerenone (*n* = 138)** | −3.57 | (−4.60 to −2.54) | 0.0051 | 2.25 | (0.75 to 3.76) |
|  | **Placebo (*n* = 123)** | −5.83 | (−6.92 to −4.73) |  |  |  |

CI, confidence interval; eGFR, estimated glomerular filtration rate; LS, least-squares.
